# SARS-CoV-2 Seroprevalence Structure of the Russian Population during the COVID-19 Pandemic

**DOI:** 10.3390/v13081648

**Published:** 2021-08-19

**Authors:** Anna Y. Popova, Viacheslav S. Smirnov, Elena E. Andreeva, Elena A. Babura, Sergey V. Balakhonov, Natalia S. Bashketova, Svetlana A. Bugorkova, Maxim V. Bulanov, Natalia. N. Valeullina, Viacheslav. V. Vetrov, Dmitriy. V. Goryaev, Tatyana N. Detkovskaya, Elena B. Ezhlova, Natalia N. Zaitseva, Olga A. Istorik, Irina. V. Kovalchuk, Dmitriy N. Kozlovskikh, Svetlana Y. Kombarova, Olga. P. Kurganova, Alexander. E. Lomovtsev, Lena A. Lukicheva, Ludmila V. Lyalina, Albina. A. Melnikova, Olga M. Mikailova, Alexei K. Noskov, Ludmila N. Noskova, Elena E. Oglezneva, Tatyana P. Osmolovskaya, Marina A. Patyashina, Natalia A. Penkovskaya, Lada V. Samoilova, Tatyana F. Stepanova, Olga E. Trotsenko, Areg A. Totolian

**Affiliations:** 1 Federal Service for Supervision of Consumer Rights Protection and Human Welfare, 127994 Moscow, Russia; anna.popova.00.00@mail.ru (A.Y.P.); ezhlova_eb@rospotrebnadzor.ru (E.B.E.); melnikova_aa@rospotrebnadzor.ru (A.A.M.); 2 Saint Petersburg Pasteur Institute, 197101 St. Petersburg, Russia; vvv-3@bk.ru (V.V.V.); lyalina@pasteurorg.ru (L.V.L.); pasteur@pasteurorg.ru (A.A.T.); 3 Rospotrebnadzor Administration in Moscow, 129626 Moscow, Russia; uprav@77.rospotrebnadzor.ru; 4 Rospotrebnadzor Administration in the Kaliningrad Region, 236040 Kaliningrad, Russia; elena_babura@mail.ru; 5 Irkutsk Research Anti-Plague Institute, 664047 Irkutsk, Russia; adm@chumin.irkutsk.ru; 6 Rospotrebnadzor Administration in St. Petersburg, 191025 St. Petersburg, Russia; nbashketova@gmail.com; 7 Russian Research Anti-Plague Institute “Microbe”, 410005 Saratov, Russia; bugorkova29@mail.ru; 8 Center for Hygiene and Epidemiology of the Vladimir Region, 600005 Vladimir, Russia; sgm@vladses.vladinfo.ru; 9 Rospotrebnadzor Administration in the Chelyabinsk Region, 454091 Chelyabinsk, Russia; ValeullinaNN@chel.surpet.ru; 10 Rospotrebnadzor Administration in the Krasnoyarsk Territory, 660049 Krasnoyarsk, Russia; goryaev_dv@24.rospotrebnadzor.ru; 11 Rospotrebnadzor Administration in the Primorsky Krai, 690950 Vladivostok, Russia; Detkovskaya_TN@pkrpn.ru; 12 Nizhny Novgorod I. N. Blokhina Research Institute of Epidemiology and Microbiology, 603950 Nizhny Novgorod, Russia; vtashca@mail.ru; 13 Rospotrebnadzor Administration in the Leningrad Region, 192029 St. Petersburg, Russia; lenobl@47.rospotrebnadzor.ru; 14 Rospotrebnadzor Administration in the Stavropol Territory, 355008 Stavropol, Russia; Kovalchuk_IV@26.rospotrebnadzor.ru; 15 Rospotrebnadzor Administration in the Sverdlovsk Region, 620078 Yekaterinburg, Russia; Kozlovskih_DN@66rospotrebnadzor.ru; 16 G. N. Gabrichevsky Moscow Research Institute for Epidemiology and Microbiology, 125212 Moscow, Russia; kombarova311@bk.ru; 17 Rospotrebnadzor Administration in the Amur Region, 675002 Blagoveshchensk, Russia; info@rospotrebnadzor-amur.ru; 18 Rospotrebnadzor Administration in the Tula Region, 300045 Tula, Russia; tula@71.rospotrebnadzor.ru; 19 Rospotrebnadzor Administration in the Murmansk Region, 183038 Murmansk, Russia; lukichevalena@icloud.com; 20 Rospotrebnadzor Administration in the Moscow Region, 141014 Mytishchi, Moscow Region, Russia; org@50.rospotrebnadzor.ru; 21 Rostov-on-Don Research Anti-Plague Institute, 344000 Rostov-on-Don, Russia; noskov-epid@mai.ru; 22 Rospotrebnadzor Administration for the Astrakhan Region, 414057 Astrakhan, Russia; tu_rpn@astrakhan.ru; 23 Rospotrebnadzor Administration in the Belgorod Region, 308023 Belgorod, Russia; orgotd@31rospotrebnadzor.ru; 24 Rospotrebnadzor Administration in the Krasnodar Territory, 350000 Krasnodar, Russia; gorses@mail.kuban.ru; 25 Rospotrebnadzor Administration in the Republic of Tatarstan, 420111 Kazan, Russia; org@16.rospotrebnadzor.ru; 26 Rospotrebnadzor Administration in the Republic of Crimea, 295034 Simferopol, Russia; crimea@82.rospotrebnadzor.ru; 27 Rospotrebnadzor Administration in the Novosibirsk Region, 630132 Novosibirsk, Russia; epid@54rospotrebnadzor.ru; 28 Tyumen Research Institute of Regional Infectious Pathology, 625026 Tyumen, Russia; StepanovaTF@Tniikip.rospotrebnadzor.ru; 29 Khabarovsk Research Institute of Epidemiology and Microbiology, 680000 Khabarovsk, Russia; trotsenko_oe@hniiem.ru

**Keywords:** SARS-CoV-2, COVID-19, herd immunity, asymptomatic form, Russia, population

## Abstract

The SARS-CoV-2 pandemic, which came to Russia in March 2020, is accompanied by morbidity level changes and can be tracked using serological monitoring of a representative population sample from Federal Districts (FDs) and individual regions. In a longitudinal cohort study conducted in 26 model regions of Russia, distributed across all FDs, we investigated the distribution and cumulative proportions of individuals with antibodies (Abs) to the SARS-CoV-2 nucleocapsid antigen (Ag), in the period from June to December 2020, using a three-phase monitoring process. In addition, during the formation of the cohort of volunteers, the number of seropositive convalescents, persons who had contact with patients or COVID-19 convalescents, and the prevalence of asymptomatic forms of infection among seropositive volunteers were determined. According to a uniform methodology, 3 mL of blood was taken from the examined individuals, and plasma was separated, from which the presence of Abs to nucleocapsid Ag was determined on a Thermo Scientific Multiascan FC device using the “ELISA anti-SARS-CoV-2 IgG” reagent set (prod. Scientific Center for Applied Microbiology and Biotechnology), in accordance with the developer’s instructions. Volunteers (74,158) were surveyed and divided into seven age groups (1–17, 18–29, 30–39, 40–49, 59–59, 60–69, and 70+ years old), among whom 14,275 were identified as having antibodies to SARS-CoV-2. The average percent seropositive in Russia was 17.8% (IQR: 8.8–23.2). The largest proportion was found among children under 17 years old (21.6% (IQR: 13.1–31.7). In the remaining groups, seroprevalence ranged from 15.6% (IQR: 8–21.1) to 18.0% (IQR: 13.4–22.6). During monitoring, three (immune) response groups were found: (A) groups with a continuous increase in the proportion of seropositive; (B) those with a slow rate of increase in seroprevalence; and (C) those with a two-phase curve, wherein the initial increase was replaced by a decrease in the percentage of seropositive individuals. A significant correlation was revealed between the number of COVID-19 convalescents and contact persons, and between the number of contacts and healthy seropositive volunteers. Among the seropositive volunteers, more than 93.6% (IQR: 87.1–94.9) were asymptomatic. The results show that the COVID-19 pandemic is accompanied by an increase in seroprevalence, which may be important for the formation of herd immunity.

## 1. Introduction

Following its appearance in late 2019, the novel coronavirus infection (COVID-19) has retained its epidemic potential for almost a year and a half. As of 31 May 2021, more than 171.2 million cases of pathogenic coronaviral infection have been registered in most countries of the world, of which more than 3.5 million (2.04%) have died. In Russia, as of 30 May 2021, there were 5,063,442 registered persons infected with SARS-CoV-2, 121,168 (2.4%) of whom have died. As these figures make clear, significant transmissibility, accompanied by a low mortality (somewhat comparable to that of influenza) is a characteristic property of this pathogen [1].

Further, although COVID-19 mortality is almost the same as that of epidemic influenza as noted, the frequency of complications (nervous, respiratory and cardiovascular systems) is much higher [2,3,4]. For a year and a half of the COVID-19 pandemic, humanity has not been able to find effective ways to treat the infection [5]. In this regard, special attention is paid to the problem of collective immunity, which is ordinarily understood as the ability to resist the pathogenic action of a specific agent (of a bacterial or viral nature) that a particular population possesses [6]. There are two ways to build resistance to the epidemic spread of any infection, including coronavirus: natural and artificial. In the former, it arises as a result of transmitted illness in susceptible individuals, as a result of their infection with a pathogenic strain. In the latter, it results from the use of specific vaccines [7]. The natural way of forming such a condition, as a result of the transmitted infection, is associated with an unacceptable risk of developing certain complications, including in some cases death [8]. In this regard, one should not expect a significant increase in the immune portion of the population which, according to contemporary estimates, might be 1–2%. This figure does not include persons who have acquired immunity to SARS-CoV-2 as a result of asymptomatic infection. Even with these cases, global population immunity does not exceed 3–6% [9].

There remains only one, highly effective way to create herd immunity: vaccination. The most impressive progress in this direction has been achieved in Israel [10]. According to these authors, by the beginning of February 2021, up to 35% of the population in that country had been vaccinated at least once, and up to 80% of these were people over 60 years old. In addition, according to the same data, about 7.5% had already had an illness and acquired immunity from infection. Thus, the overall prevalence of collective immunity exceeded 42%. As a result, according to the authors, the country managed to achieve a 100-fold decrease in the incidence rate and a 50-fold decrease in the number of severe cases in 90 days [10]. With regard to collective immunity in other regions, the highest levels were recorded in Iran, Sweden and Chile. The lowest were in Greece, Malaysia, and Brazil [9].

The effectiveness of herd immunity formation, natural or artificial, depends on a threshold level which, in turn, is determined by the degree of susceptibility of individuals and the population as a whole, and the intensity of contacts (and the associated probability of transmission of the pathogenic agent to susceptible individuals). The number of secondary infections that one infected person can cause, in a fully susceptible population, is called the base reproductive number (R_0_) [7]. With regard to SARS-CoV-2, this value is about 5.7, which corresponds to a herd immunity level of 82.5% [11]. Such calculations make it possible to predict more accurately the rate of formation of population immunity, one of the essential characteristics of which is the formation rate of specific antibodies.

Serological studies are an important tool for epidemiological monitoring of the spread of infection, identification of risk groups and the development of adequate measures to control the spread of infection. Modern sero-monitoring is based on determining the quality and quantity of antibodies (Ab) specific to a virus or its individual (Ags). Such detection makes it possible to assess not only pathogen contact, or response levels to the introduction of specific vaccines, but also to assess a person’s degree of protection from development of the disease [12]. In addition, such assessment is indispensable for retrospective identification of asymptomatic cases, as well as for identifying people who have recovered from COVID-19, whose antibodies can be used for plasma therapy for critically ill patients [13].

Since the beginning of the COVID-19 pandemic, many researchers have focused on obtaining global and regional sero-epidemiological information [9,14,15]. In Russia, the first studies of collective immunity in the population to SARS-CoV-2 were launched at the initiative of the Federal Service for Supervision of Consumer Rights Protection and Human Welfare (Moscow, Russia) with the participation of the St. Petersburg Pasteur Institute. (St. Petersburg, Russia) The project is being implemented in three phases. The first phase is a cross-sectional study of SARS-CoV-2 seroprevalence in 26 model regions of Russia. The second and third phases are a longitudinal study of seroprevalence dynamics in cohorts of volunteers, formed in model regions in the first phase. Considering that a three-phase study was carried out in 30.5% of all constituent entities of the Russian Federation, wherein live 84,944,131 (58.1%) individuals, the results obtained can quite correctly be extrapolated for the entire Russian territory. The purpose of this work was to analyze and summarize data on the state of Russian population immunity to SARS-CoV-2 during the COVID-19 pandemic in 2020.

## 2. Materials and Methods

### 2.1. Study Organization and Formation of a Volunteer Cohort

The first studies of collective immunity to SARS-CoV-2 in Russia were launched in June 2020, simultaneously in 26 regions of the country, within the framework of the program “Assessment of population immunity to the SARS-CoV-2 virus in the Russian population”, in the context of the COVID-19 pandemic, developed by the Federal Service for Surveillance on Consumer Rights Protection and Human Welfare (Rospotrebnadzor, Moscow, Russia) with the participation of the St. Petersburg Pasteur Institute (St. Petersburg, Russia), taking into account WHO recommendations [16]. The study was approved by the local ethics committee of the St. Petersburg Pasteur Institute (Protocol No. 64, dated 26 May 2020). Prior to the study, all the participants or their legal representatives were informed about the purpose and methods of the study; all of them signed the informed consent. The exclusion criterion was active COVID-19 infection at the time of the survey.

To conduct research on the population’s immunity to SARS-CoV-2 in each of the 26 regions of Russia, a group of volunteers was recruited, consisting of at least 2688 people. Selection was carried out by the method of online survey using cloud (internet server) technologies and randomization by age and area of residence. A total of 74,128 people were selected, which amounted to 2802 people (95% CI 2588–2938) in each region. All volunteers were divided into seven age groups (years old): ‘1–17’; ‘18–29’; ‘30–39’; ‘40–49’; ‘50–59’; ‘60–69’; and 70 or above (‘70+’). In addition, to take into account the dynamic processes involved with pediatric immune system maturation, the children’s cohort was divided into three age subgroups (years old): ‘1–6’; ‘7–13’; and ‘14–17’.

The regional principle of randomization assumed an equal distribution of volunteers in the population centers of each of the surveyed areas of Russia. A limitation was the number of participants in the study. There could be no more than 30 from one organization. This excluded participation in the study by organized groups (preschool institutions, schools, military units, etc.). Cohort sizes for each region and age distribution are presented in Table S1 [17].

Thus, selection of volunteers and randomization according to regional and age characteristics made it possible to obtain a generally homogeneous set of samples in each district of the Russian Federation. Although the number of subjects examined in each group varied, these variations were generally small. The total number of outlier values, due to volunteer response characteristics, did not exceed 5%, and they do not affect the main trend represented by median, upper and lower quartiles for 2802 (IQR: 2688–2938) people.

Analysis of serological prevalence dynamics (Abs to SARS-CoV-2) was carried out in three phases: the first phase (volunteer cohort formation) in June–July 2020; the second phase in September to October 2020; and the third phase in December 2020. The same participants were examined in all phases. The first phase of sero-monitoring was combined with the formation of an initial cohort of volunteers. Most of the volunteers examined during cohort formation participated in serological examination in the second and third phase of sero-monitoring (Table S2).

### 2.2. Antibody Analysis Methodology

The main objective of the study was to assess the serological prevalence of Abs to the nucleocapsid Ag (Nc) of SARS-CoV-2 among the Russian population. For this, 3 mL of venous peripheral blood was collected from volunteers into vacutainers with EDTA and centrifuged. Plasma was separated from cellular elements and stored at +4 °C until analysis. Plasma samples were analyzed by enzyme immunoassay on a Thermo Scientific Multiascan FC (Vantaa, Finland) device for the presence of specific type G immunoglobulins to SARS-CoV-2 Nc using a reagent kit (prod. Scientific Center for Applied Microbiology and Biotechnology, Obolensk, Moscow Region, Russia) according to the manufacturer’s instructions [18,19].

### 2.3. Statistical Analyses

Statistical processing was carried out by methods of non-parametric statistics using Excel and WinPepi software (version 11.65). Confidence intervals for proportions were calculated according to A. Wald’s method, with correction according to Adresti and BCoul [20], using a special calculator (https://measuringu.com/calculators/wald/) (accessed on 1 June 2021), as presented [21]. In a number of studies, the standard error of the share was determined with this resource (https://statanaliz.info/statistica/opisanie-dannyx/dispersiya-i-standartnaya-oshibka-doli/) (accessed on 9 June 2021). Normality of distribution was determined according to the Kolmogorov-Smirnov goodness-of-fit test (https://medstatistic.ru/methods/methods10.html) (accessed on 9 June 2021). Since the distribution was different from normal, the median (Me), lower (Q25) and upper (Q75) quartiles were calculated. Correlation dependence was estimated by the Spearman method. In paired linear regression, the following equation was obtained: y = a0 + y a1 × x, where a0 and a1 are the constant and the regression coefficient, and x is the value of the dependent variable. To assess the quality of the selection of a linear function, the determination coefficient R^2^ was calculated. A determination of ‘statistically significant’ (differences in indicators) was applied when *p* ≤ 0.05.

### 2.4. General Features of Collective Immunity to SARS-CoV-2 in the Russian Population

An essential condition for the implementation of the program for studying the collective immunity to SARS-CoV-2 among the population of Russia was the formation of a cohort of volunteers representative of the entire country’s population. For this purpose, model regions were selected, representing all Russian FDs, in such a way that the overall cohort was representative of the total population (Table 1).

After selection of volunteers in 26 regions of the country, the entire cohort was examined for the presence of anti-Nc antibodies. Subsequent analysis of the results obtained for the FDs showed that more than 50.8% of the Russian population live in the area of the 26 surveyed regions (Table 1). 

The size of the selected cohort of volunteers corresponds to the volume required to reach the threshold of representativeness [34]. The largest number of regions was surveyed in the Central, Southern, and Northwestern FDs. After grouping the regions by FD, it was found that the average level of seroprevalence among the population, among volunteers of all ages without age stratification, is 19.2% (95% CI: 18.9–19.5). 

The highest levels of seropositivity were found in the Northwestern FD (31.9% (95% CI: 30.9–32.9)) and the Far Eastern FD (28.7% (95% CI: 27.8–29.7)). The lowest were found in the North Caucasus FD (9.8% (95% CI: 8.6–11.0)) and the Siberian FD (9.2% (95% CI: 8.3–9.7)). In all cases, the differences were statistically significant (*p* < 0.05). 

Comparison of the obtained results with literature data showed that seroprevalence in Russia is 3.5-fold higher than the world average (3.38% (95% CI 3.05–3.72)); 5.7-fold higher than in Western Europe (3.17% (95% CI 1.96–4.38)); and more than 4-fold higher than in the U.S. (4.41% (95% CI: 3.03–5.79)) [9,35].

Thus, the developed methodology made it possible to obtain a representative idea of the seroprevalence level of the Russian population in the early period of the COVID-19 pandemic (June–August 2020). As we see, the largest proportion of seropositive persons was found in coastal regions of the Northwestern FD and the Far Eastern FD, characterized by moderately humid, cool climates with a high level of industrialization. Further, the minimum share of seropositive volunteers was found the North Caucasus FD (represented in the project only by the Stavropol region, mainly of an agricultural orientation) and the Siberian FD (with a sharply continental climate and a predominance of the mining industry).

Moreover, the FDs, due to Russia’s vastness, turned out to be rather heterogeneous in climatic/geographical conditions, population density, and industrial specialization. In this regard, the data presented in Table 1 provide only a general picture. A more detailed understanding is enabled by analysis of seroprevalence in individual Russian regions (administrative districts, municipalities).

### 2.5. Territorial and Climatic Features of Collective Immunity to SARS-CoV-2 in the Russian Population

The territory of the Russian Federation is located in several climatic zones, from the subtropics (Southern FD) to the circumpolar belt (territory beyond 60 degrees north latitude of the Northwestern FD). As such, a significant impact of climatic and geographic conditions on the formation of collective immunity in the population is inevitable. In addition, population densities have a definite impact. The highest are in the Southern FD, the Central FD, and a number of areas in the Northwestern FD. The lowest densities are in the Siberian FD. Finally, the age factor is also of no small importance. It is quite obvious that collective immunity formation dynamics among the population will be significantly different.

The average seropositivity value among the Russian population was 17.8%; (IQR: 8.8–23.2). The range of variation across regions was 10.7-fold (Table S3). The highest level of percent seropositive was found in the Kaliningrad region (50.2% (95% CI 48.4–52)), and the lowest was in the Republic of Crimea (4.3% (95% CI 3.6–5.1)). These regions are located in different geographical areas. The Kaliningrad region is located at 54°42′23″ north latitude in a zone of moderately humid, cool climate. St. Petersburg and the Leningrad Region are in the same climatic zone. The Republic of Crimea is located between 44°23″ (Cape Sarych) and 46°15″ (Perekop canal), in three climatic zones: steppe; mountainous; and Mediterranean. As for other regions, due to the country’s vast area, there are those with a monsoon climate (Primorsky region) and a sharply continental cold climate (Irkutsk and Novosibirsk regions, the Middle Urals area). A zone in the Central FD features a moderate, cool climate (Moscow and Moscow region, Vladimir region). Finally, southern regions feature warm winters and hot summers (Rostov and Krasnodar regions).

**Figure 1:**
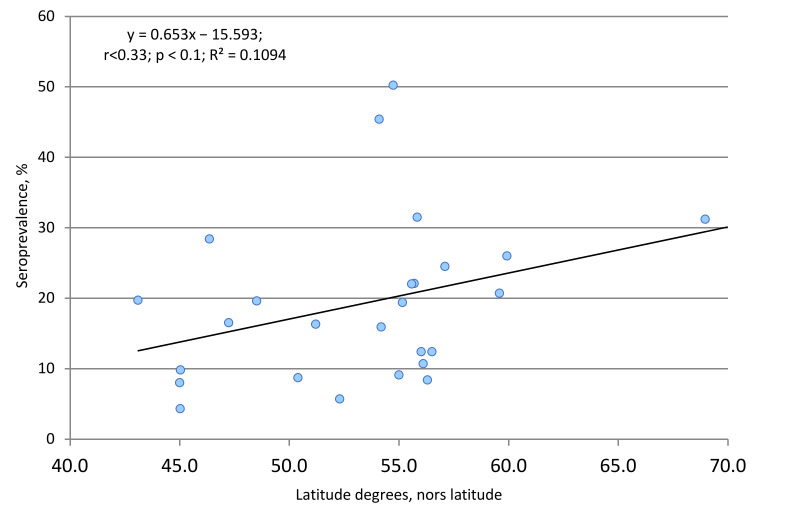
Correlation between geographical latitude of region and percent seroprevalence. The equation, regressions, correlation coefficient (r), statistical significance level (*p*), and determination coefficient (R^2^) are shown. Geographic latitude is given for the center of the region.

Naturally, on such a large territory, one can only outline the climatic features of a particular region. Significant differences in climatic and geographic characteristics could have some effect on Ab generation in the local population. At first glance, such a relationship exists (Figure 1). In the Spearman correlation analysis, the value (r) was 0.33, and the critical value of the coefficient was 0.33 at *p* < 0.1. Thus, a weak, direct correlation was found between percent seropositivity and latitude. It, however, did not reach the recognized confidence threshold (*p* = 0.05).

Another factor that can influence infectious disease spread and collective immunity level is population density. Unfortunately, it was not possible to establish such a relationship between the population density of a particular category and the level of population immunity. Spearman’s correlation coefficient was 0.14 without taking into account the population density of Russia’s largest entities (Moscow city, St. Petersburg city, and the Moscow region). Taking these regions into account, the correlation coefficient decreased to 0.12.

A possible reason may be the mosaic settlement of regions, characteristic of Russia. This implies that, in the first six months of the pandemic, seroprevalence was formed mainly due to random contact between individuals. The validity of this assumption was tested by attempting to identify a relationship between seroprevalence and the incidence rate. To determine the presence of a relationship between these indicators, we compared the percent seropositive individuals in each region and the incidence rate, which is the average value of the daily incidence during the period of blood sampling for serological research. Determination of the rank correlation value according to Spearman did not reveal a significant relationship between the compared indicators. The coefficient r value was 0.22, and the critical value r was 0.33 at *p* > 0.1; the relationship is statistically insignificant.

Among the factors that can significantly affect the collective immunity formation rate, population age structure can have a significant impact. It is known that immune processes occurring in childhood and old age differ significantly from each other. Children most often carry the illness in an asymptomatic form or as a respiratory infection (common cold) with nonspecific symptoms, such as fever, cough, myalgia, or fatigue [36,37]. In most cases, the illness is milder than in adults and most often it resolves well. A different situation occurs with older ill individuals or seniors (70+). In most cases, this category of people suffers from one or more chronic, age-related diseases, such as diabetes mellitus, chronic heart and/or pulmonary failure, angina pectoralis, atherosclerosis, or other age-related diseases; these inevitably complicate the course of COVID-19 [38,39]. In this regard, it was logical to expect that volunteer age could have a certain influence on the formation of population immunity. The general distribution of the proportion of seropositive volunteers, of all ages in 26 model Russian regions, is presented in Table S2.

**Figure 2:**
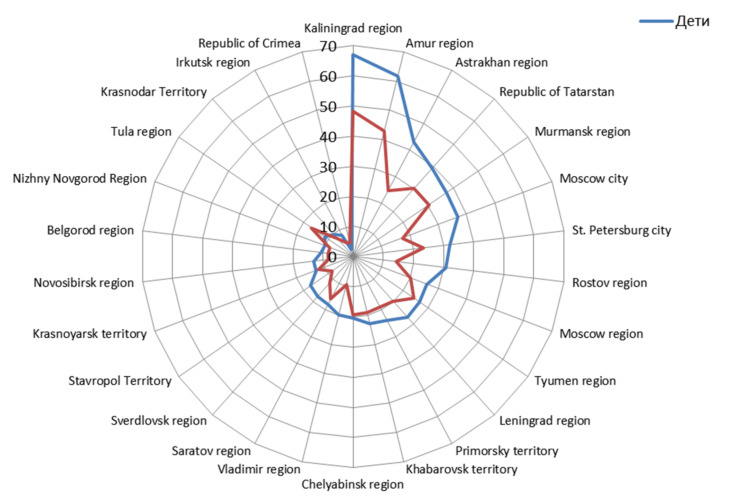
Distribution of seropositive volunteer proportions in the child cohort (blue curve) and adult cohort (red curve). The vertical axis is the percent seroprevalence (SARS-CoV-2).

Analysis of volunteer distribution by age showed a wide spread in the percent seropositive from region to region. In 23 out of 26 regions, the highest levels were observed in ‘children aged 1–17 years’. Only in three (Republic of Crimea, Tula and Chelyabinsk regions) was the proportion of seropositive persons among volunteers less than in other age groups (Figure 2).

Increased resistance to SARS-CoV-2 is considered a characteristic feature of children aged 1 to 17 years [9,22,40] There are several explanations for this phenomenon. First, the existence of cross-immunity to other β-coronaviruses, weakly pathogenic for humans, has been shown, in particular to HCoV-OC43 and HCoV-HKU1 [41,42]. In addition, children show lower expression of the ACE-2 gene than adults. The early age period is characterized by the smallest expression, below 2.0 log_2_. From 10 to 25 years, gene expression increased from 2.40 log_2_ to 3.09 log_2_ (*p* < 0.001). Of these two explanations, preferable is that of Ng et al. [40], who showed that: widespread, mildly-symptomatic cold-like illnesses, caused by a list of weakly pathogenic viruses, can induce the formation of antibodies to the SARS-CoV-2 S2 Ag in a child’s body, even in the absence of SARS-CoV-2 contact. Once in children, these Abs can be retained in adults, albeit at a lower concentration.

**Figure 3:**
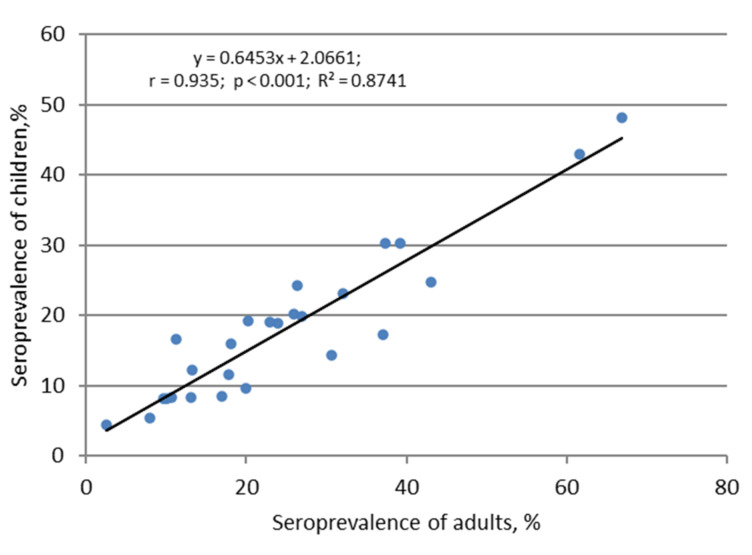
Correlation relationship between seroprevalence in children and adults. The regression equation, Spearman correlation coefficient (r), reliability of statistical relationship (*p*), and the coefficient of determination R^2^ are shown.

In addition, pediatric SARS-CoV-2 infection often proceeds in a latent, asymptomatic form, in which the infectious process remains unrecognized, and the fact of the transferred infection can only be judged by the presence of circulating Abs to SARS-CoV-2 [43]. As shown by Hippich et al. [44], the real incidence of SARS-CoV-2 infection is six-fold higher than the number of reported cases in children. Moreover, the general dynamics of antibody formation follows the general pattern of infectious process development in the population as a whole. This conclusion is prompted by the seroprevalence distribution curves in Russian children and adults (Figure 2) and is confirmed by the correlation between the levels of seroprevalence in children and adults (Figure 3).

**Figure 4:**
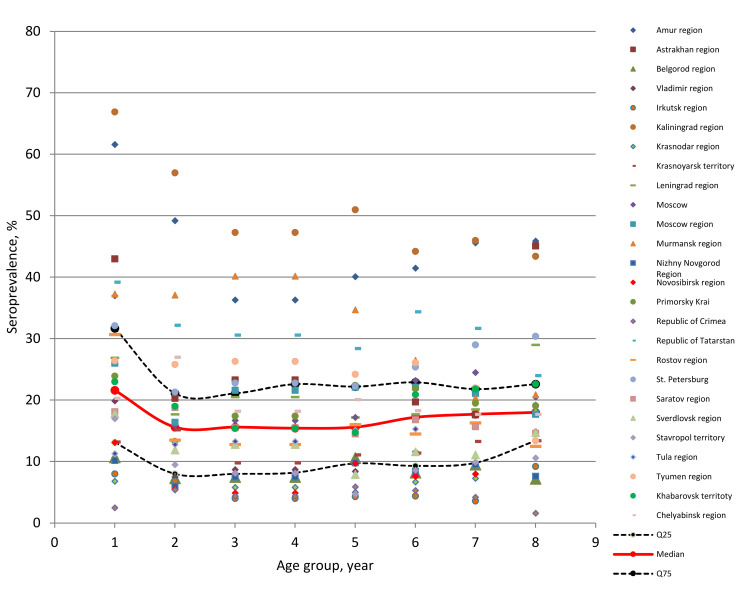
Distribution of volunteer seroprevalence by region and age. Solid curves correspond to: Me (median); Q25 (bottom); and Q75 (top). Dots indicate volunteer seropositivity values in 26 model regions (legend), distributed over 7 age groups.

The analysis results showed a practically functional correlation between the compared values of seroprevalence in children and adults (Figure 3). With regard to the dependence of the seropositivity level among children and adults from 18 to 70 years and more, divided into six age categories, among a number of age groups in some regions, a statistically significant increase or decrease in percent seropositivity was observed in comparison with the final data (Table S3). These differences are almost completely leveled out when calculating the median and IQR for each age group within the entire set of regional cohorts (Figure 4). As seen in Figure 4, 75% of the ‘proportion of seropositive volunteer’ values, in all age groups, are located within the IQR. Accordingly, 12% of the seropositivity points (from the entire data above) are located above and below the IQR.

The results above give reason to believe that, during formation of volunteer cohorts for follow-up, the proportions of seropositive individuals were relatively evenly distributed, both by age and model region. Taking into account that similar trends were revealed when grouping the results by Russian FDs (Table 1), it can be reasonably assumed that the revealed seroprevalence (SARS-CoV-2 Nc) is typical for the Russian population as a whole. Its main features can be considered an increase in the level of seropositivity in ‘children aged 1–17 years’ (Figure 2) and a weak dependence of seroprevalence on the latitude of the northern hemisphere (Figure 1).

### 2.6. Three-Phase Monitoring of SARS-CoV-2 Seropositivity

During initial examination of volunteers, data were obtained shown a moderate distribution of the proportion of seropositive individuals across Russia (Table S3). The average seropositivity was 19.2% (95% CI: 19.0–19.6). Moreover, the differences between FDs turned out to be statistically significant and consisted of distributions between individual Russian regions. In this regard, a dynamic observation was carried out on the (proportional) formation of seropositive volunteers, in all model regions, from September to December 2020, during the period in which the second wave of COVID-19 incidence was observed (https://coronavirus-monitor.ru/, accessed on 10 June 2021).

In the first phase of monitoring, cohorts were formed from the selected groups of volunteers, which were subsequently observed in the second and third phases of serological study (Table S2). For various reasons in each phase, the number of volunteers involved in monitoring varied.

**Figure 5:**
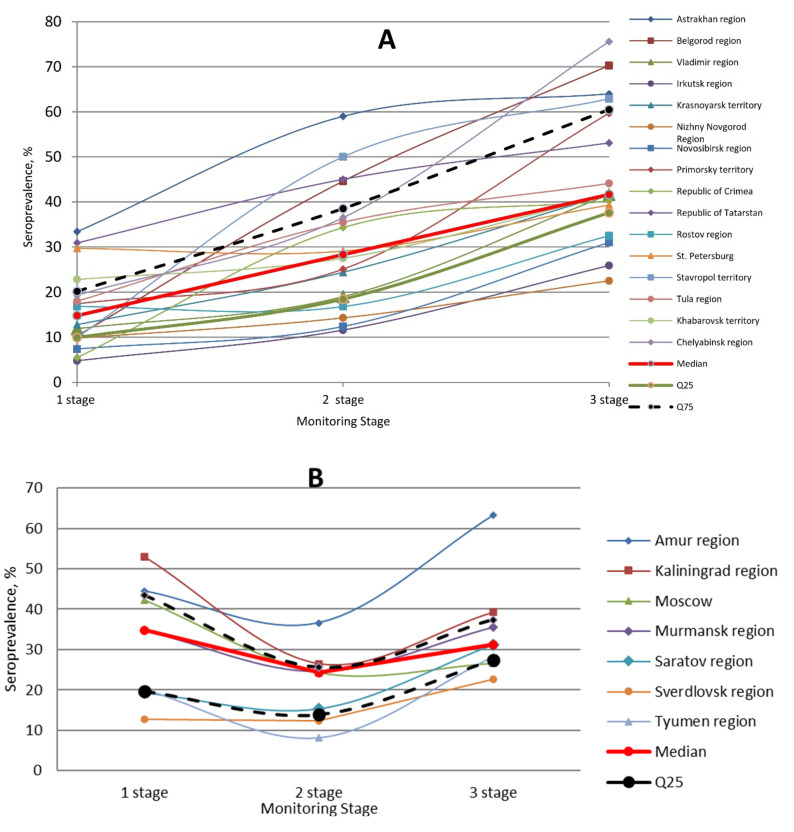

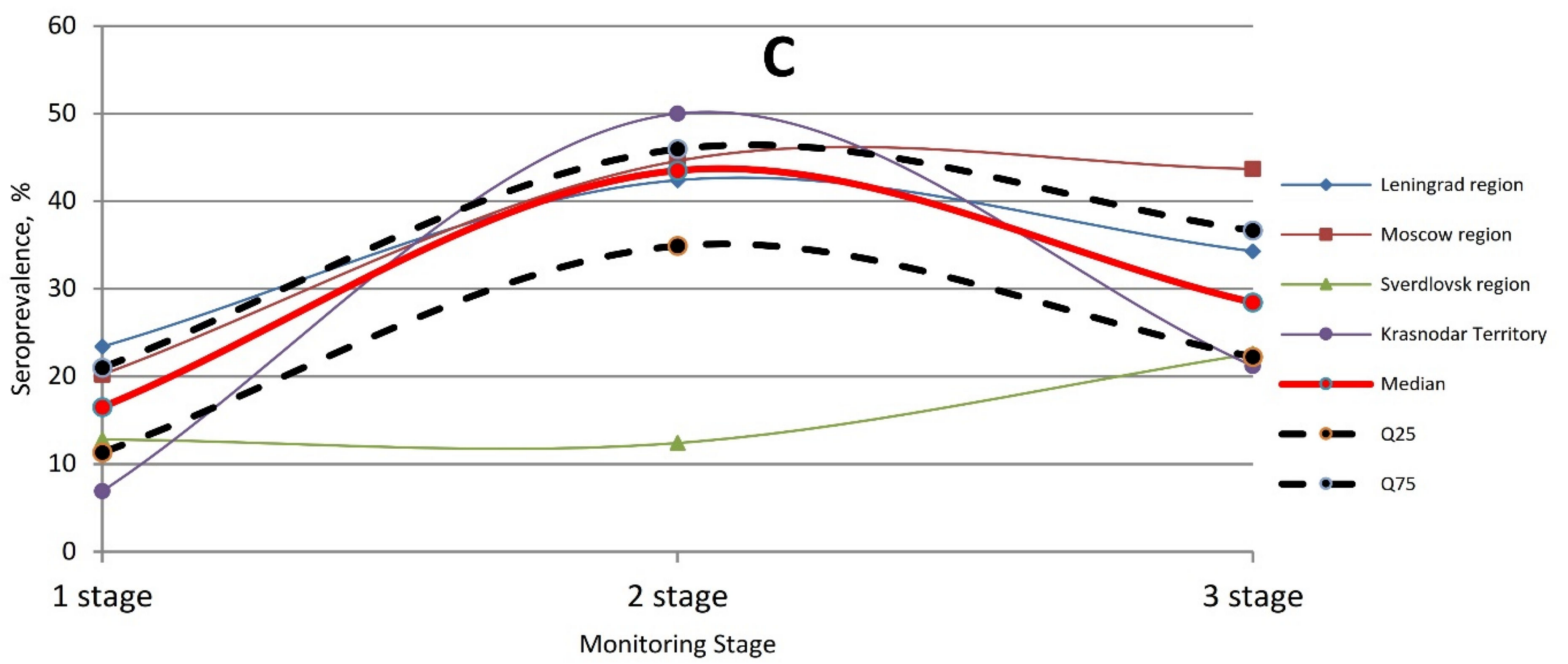
Three curve types for the formation of population immunity to SARS-CoV-2 in different Russian regions. (**A**). The most common dynamics of formation of specific SARS-CoV-2 immunity. In the process of population contact with coronavirus, the proportion of seropositive individuals in the population increases, reaching a maximum by the 3rd phase. (**B**). Less frequent curve showing a slower process of seropositivity formation. By the 2nd phase, the percent of the Nc Ab seropositivity volunteers decreased. The maximum Ab level is formed only by the 3rd phase. (**C**). The rarest paradoxical reaction in which percent seropositive increases between phases 1 and 2, and then decreases markedly. Immunity formation dynamics in the surveyed regions are shown by thin colored lines. The median is represented by a thicker red curve. The boundaries of the interquartile range Q25—Q75 are plotted as bold, dashed lines.

Seropositivity formed at the beginning of the study (phase 1) amounted to 18.6%, (IQR: 10.7–23.0). In phases 2 and 3, it increased to 27.4% (IQR: 17.4–38.0) and 39.8% (IQR: 31.0–43.8), respectively (Figure 5). The differences between the first and third phase results are statistically significant (*p* < 0.05). Depending on the type of response, the proportion of seropositive individuals could vary significantly. In group A, which includes 16 regions, seropositivity unidirectionally increased from 14.8% (IQR: 10.0–20.2) in phase 1 to 41.7% (IQR: 37.6–60.5) in phase 3, (*p* < 0.05). In group B, which contained seven regions, the changes were different. In the 1st phase, the proportion seropositive was 34.8% (IQR: 19.6–43.4). In the second, it decreased to 24.3% (IQR: 13.9–25.4), and by the third phase, it had increased to 36.2% (IQR: 27.4–37.4). Group C includes only three regions. In the first phase, the proportion seropositive was 20.2% (IQR: 13.5–21.8). By the second phase, it had increased to 44.6% (IQR: 43.5–47.3) (*p* < 0.05). In the third phase, it again dropped to 34.3% (IQR: 28.8–39).

In some areas, the proportion of seropositive individuals came close to the minimum proportion of seropositivity at which intrapopulation transmission of infection ceases. According to the literature, it is: 68.6% (IQR: 45.4–72.9) [45]. The closest to the given threshold came (regions): Chelyabinsk region (75.6% (95% CI 73.3–77.9)); Belgorod region (70.3% (95% CI 68.7–72.5)); Amur region (66.3% (95% CI 64.3–68.3)); Astrakhan region (64.0% (95% CI 61.3–66.6)); and the Stavropol region (62.9% (95 % CI 59.0–65.9)). It is worth noting that studies in the third phase were carried out in the second half of December 2020; already in the first two weeks of January 2021, in most of the surveyed regions, there was a steady trend towards a decrease in incidence, which continued until the end of May 2021 (https://coronavirus-monitor.ru/, accessed on 10 June 2021) It is likely that the collective immunity of the population formed by the end of 2020 had a positive effect on incidence trend changes, although the average seropositivity rate in Russia as a whole did not reach the required level by the end of 2020; it amounted to only 39.8% (IQR: 31.1–43.8). This is largely due to heterogeneity in the seropositivity accumulation process. In addition to the regions already listed above with a high seroprevalence threshold value, regions were noted wherein the number of carriers of antibodies remained quite low. Specifically, these included: the Krasnodar region (21.2% (95% CI 17.1–23.1)); the Sverdlovsk region (22.6% (95% CI 19.3–24.5)); and the Irkutsk region (25.5% (95% CI 22.7–27.9)). In a number of regions, however, the initial seroprevalence level in the first phase did not exceed 5%–7%, yet in the process of epidemiological observation of the surveyed volunteers, the level of immunity rose by three- to four-fold.

Summarizing the results of three-phase monitoring, we note that, in the process of epidemiological observation, a significant increase in collective immunity was shown in all surveyed regions. In some areas, it reached or was close to the minimum protective threshold [7,9,45]. In other cases, it was quite low, although in some cases it significantly increased (relative to the initial level noted in the first phase). There was not a single case of a decrease in SARS-CoV-2 seropositivity in the dynamics of sero-monitoring (Figure 5).

Unfortunately, the trend towards a decrease in COVID-19 incidence was observed only at the beginning of June 2021 (https://coronavirus-monitor.ru/, accessed on 10 June 2021) In the first week of June, the trend reversed and was replaced by a daily increase in incidence, which began at a higher level than before the second peak (observed in September–December 2020). It should be noted that, in May 2021, widespread vaccination began. In this regard, it is rather odd to record another rise in incidence, very reminiscent of a beginning of a third COVID-19 wave. An obvious dissonance in these conditions was made by S. S. Sobyanin on 18 June 2021, as given to Lenta.ru, on a decrease in the level of population immunity in Moscow from 60% to 25% (https://lenta.ru/news/2021/06/18/sobanin/, accessed on 18 June 2021). If these data are correct, then science faces many questions regarding the duration and effectiveness of SARS-CoV-2 immunity.

### 2.7. Seroprevalence among COVID-19 Convalescents and Contacts

**Figure 6:**
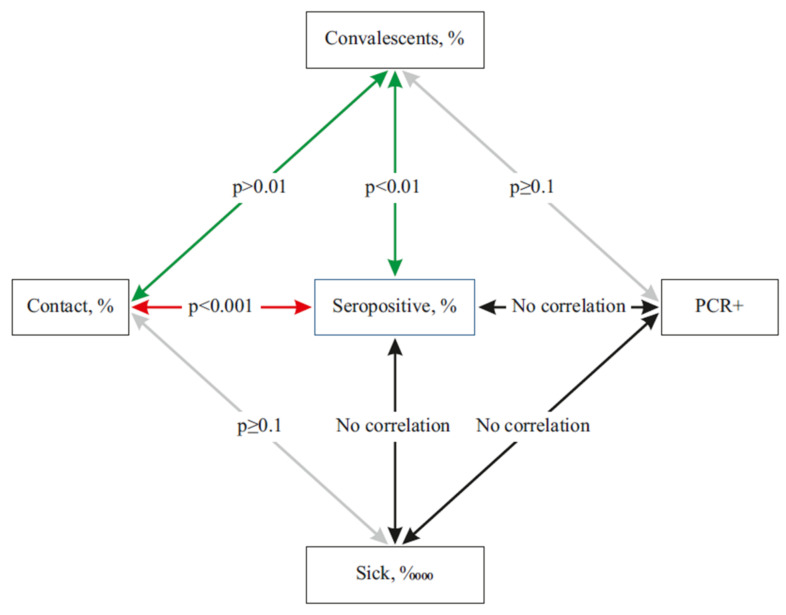
Spearman correlations between COVID-19 patient states. The diagram contains five main patient conditions, which describe basic features of the interaction between the coronavirus and a susceptible host. Key: seroprevalence—the proportion of seropositive individuals in the population according to ELISA data; patients—infected with SARS-CoV-2 based on PCR results and, as a rule, having clinical manifestations of COVID-19; convalescents—patients who have recovered from COVID-19, based on PCR results; contact—persons who have had verified contact with a COVID-19 patient; PCR^+^—persons with a positive PCR test result without any other clinical manifestations. Colored lines mark reliable correlations between the indicators. Weak correlations are marked with gray lines; black lines indicate an absence of correlation. Reliability of relationship values (*p*) are shown on the arrows.

Among people who are SARS-CoV-2 seropositive, persons who have undergone a clinical form of COVID-19 and persons who have had contact with patients can play certain roles in the epidemic process. Among the latter, one can most often find employees of medical institutions [46]. Finally, a special place in the transmission and immune processes is played by asymptomatic seropositive individuals, both with and without positive results from viral RNA testing by polymerase chain reaction (PCR^+^). That (last) population category causes a particularly active discussion [47,48]. There are different views on the relationships between indicators of the infectious process (Figure 6). 

The diagram in Figure 6 shows a certain logic in virus/host interaction processes, and also clearly demonstrates the complexity of the connections, which do not always fit into the Procrustean bed of correlation analysis. It would seem that there is an obvious functional relationship between the number of cases and the proportion of seropositive cases. However, morbidity is recorded at the time of a patient’s visit to a medical or prophylactic institution, while the formation of seroprevalence occurs in a later period [49,50]. Thus, it can be assumed that the dynamics of these processes will be different. It is also important to determine the relationship between the proportion of seropositivity in PCR^+^ individuals and the incidence rate. As a rule, it is impossible to assess the relationship between PCR-positivity, the presence of anti-Nc (SARS-CoV-2) antibodies, and COVID-19 diagnosis. These processes develop independently of each other, and it is probably inappropriate to look for some kind of correlation between them.

**Figure 7:**
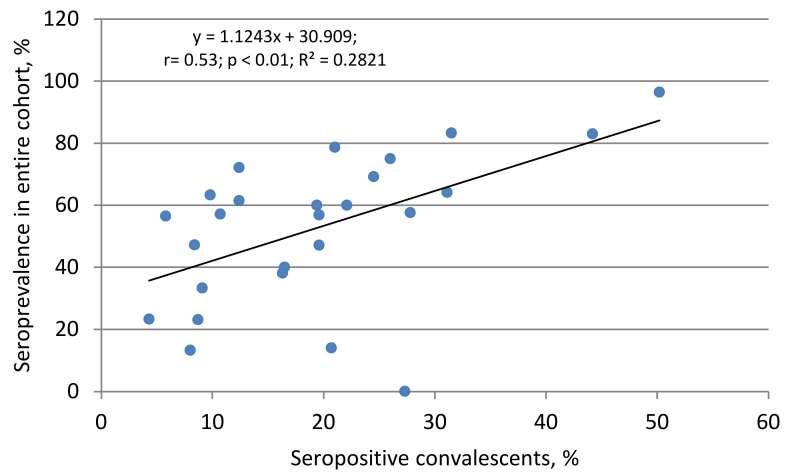
Correlation between seropositivity in the cohort as a whole and the proportion of seropositive among convalescents. The equation, regression, Spearman correlation coefficient (r), the reliability of relationship (*p*), and the coefficient of determination are given.

The links between seropositivity in general, and separately in convalescents and people who have been in contact with coronavirus patients, are clearer. A direct correlation was established between seropositivity in the cohort as a whole and the proportion of seropositive convalescents (Figure 7).

**Figure 8:**
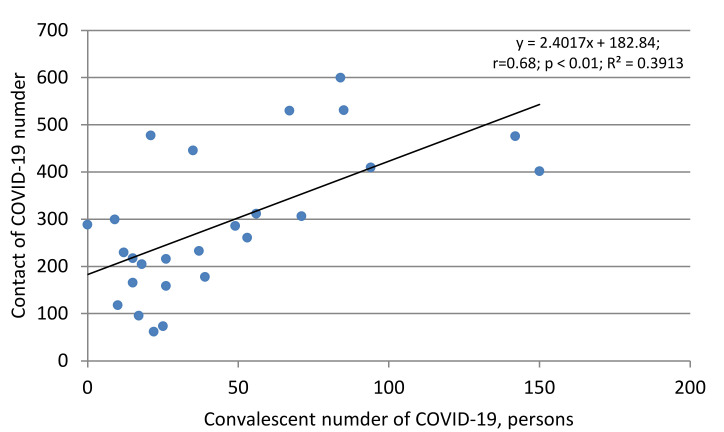
Correlation between the number of convalescents and COVID-19 contacts. The equation, regression, correlation coefficient value (r), reliability relation (*p*), and the coefficient of determination (R^2^) are shown.

It is clear that, in the general cohort, the proportion of seropositive individuals with verified contacts with COVID-19 patients is one of the components of the overall level of collective immunity. It seems more significant to study the relationship between the number of COVID-19 patients and those who have had verified contact with them at work or at home. There is ample evidence that the virus is highly contagious. According to various sources, one patient, or even one who has been ill (yet in the early period after clinical recovery), can be a carrier of the virus; such people are capable of infecting from one to ten or more healthy individuals, depending on the intensity of contacts and the availability of personal protective equipment [7,48]. Obviously, the number of contact persons in these conditions can be associated with specific connections and serves as a factor in the spread of infection. Indeed, the number of contact persons is directly correlated with the number of convalescents (Figure 8).

The revealed relationship confirms that there is a stable correlation between the compared indicators, with a reliability of *p* < 0.01. The ratio between the compared indicators, which can be considered the baseline R_0_ value without adjustment for seropositivity, was from 1.4 to 6.9 [51]. This indicator is approximate, since it does not take into account the effectiveness of the contact, accompanied by an immune response in the form of antibody production against SARS-CoV-2. After adjusting for seroprevalence, R_0_ was 2.0.

**Figure 9:**
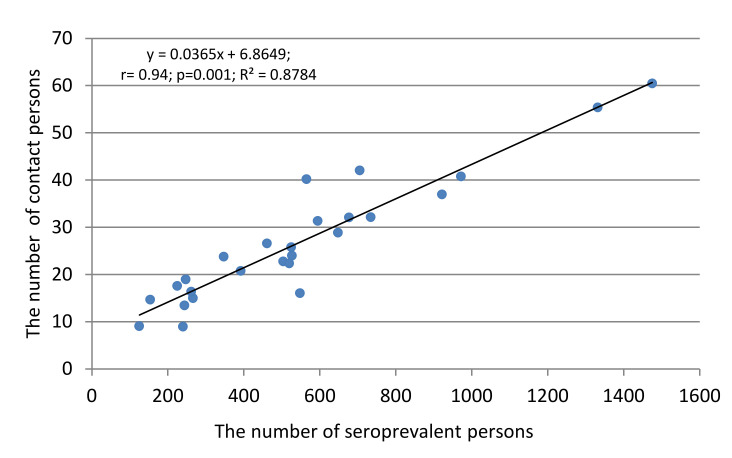
Correlation between the number of seropositive and COVID-19 contacts. The equation, regression, correlation coefficient value (r), the reliability of the relationship (*p*), and the coefficient of determination (R^2^) are shown.

The third factor influencing the course of the epidemic process may be the ratio between contact and seropositive persons. A special feature of COVID-19 is the difficulty in contact tracing. A contact is not necessarily accompanied by a clinical or even serological response. On the other hand, a pre-symptomatic or asymptomatic contact subject may be an active spreader of the virus in a susceptible population [52]. Within the framework of this study, the presence of a relationship between the proportion of seropositive persons among contact persons and the cohort as a whole was assessed. Initially, we calculated the correlation coefficient between the absolute number of seropositive and contact persons in each region (Figure 9).

The results show that the number of seropositive and contact persons are connected with each other by a practically functional relationship with a degree of reliability exceeding *p* = 0.0001. This, in our opinion, convincingly indicates that persons who have had verified contact with COVID-19 patients are actively involved in the formation of population immunity, although contact between a healthy person and a COVID-19 patient is not always accompanied by the manifestation of an infection caused by contact transmission from the patient to the healthy individual.

### 2.8. Asymptomatic SARS-CoV-2 Infection

A characteristic feature of coronaviral infection is a large number of asymptomatic individuals [53,54]. It is known that those with asymptomatic forms of infection not only do not significantly help in the formation of herd immunity, but often actually pose a risk (by participating in pathogen transmission and spread) [55]. As part of the program for assessing population immunity to SARS-CoV-2, seropositive volunteers who did not have any symptoms of an overt illness were classified as asymptomatic individuals. Among 14,158 seropositive for Nc SARS-CoV-2 Ab volunteers, 13,026 (19.2%) such participants were identified with asymptomatic forms of COVID-19. The distribution of such individuals by regional and age characteristics turned out to be quite homogeneous (Table S4). The weighted average across the entire cohort was 93.6% (IQR: 87.1–94.9). In 12.1% of groups, the number of asymptomatic people reached 100%. Most often, a completely asymptomatic course was observed among people in the 70+ group (30.7%), and less often in the 60–69 age group (3.8%).

Thus, the near absolute frequency of seropositive, asymptomatic individuals is 17.7% of the total number of volunteers. Is this high or low? From the herd immunity standpoint, it is small, if we consider an optimal threshold of 60–65% seroprevalence [7]. Concerns about their possible role in viral transmission, or maintenance of the epidemic process, are numerous. Even if we take into account a not very high R_0_ value (2.0 in our studies), 13,036 asymptomatic individuals can still become a source of infection for up to 35,000 new cases (without taking into account a multiplier effect). In this regard, it is appropriate to recall the data of Khoshchehreh et al. [56], who showed that Nc-positive individuals are often seropositive, but not necessarily protected from SARS-CoV-2 infection [57]. At a minimum, these data once again convincingly indicate the most important task of managing the COVID-19 pandemic: the fastest possible formation of herd immunity which, given the conditions present in Russia, should be at least 60%–65%. There is only one way to reach this level: by total vaccination of the population.

## 3. Discussion

Serological studies occupy an important place among the means and methods of controlling the novel coronavirus disease, COVID-19. They have not stopped being informative or relevant, despite the introduction of new means and methods for controlling emerging outbreaks, epidemics, or even pandemics of infectious diseases [9,58,59]. Sero-monitoring is the most convenient tool for enabling objective assessment of the direction and dynamics of the epidemic process, more effective and timely decisions aimed at controlling the infectious process, and the formation of population immunity [6,45]. Currently, a large number of works, of varying quality, have been published on the problem of population immunity to SARS-CoV-2, as summarized in a number of overview articles and meta-reviews [9,47,60]. Published works on population seroprevalence in a number of countries or regions have been provided: China [61]; Israel [10]; the Near and Middle East [9,62,63]; most countries of Europe [60]; North and Latin America [64,65]; Australia [66]; and Russia [17,22].

According to generalized statistics, seroprevalence among men was 5.33% (95% CI 4.35–6.31), with 5.05% (95% CI 4.06–6.04) among women. A significantly higher seroprevalence was noted in Qatar, with 66.8% (95% CI 65.4–68.1) among men, and 18.5% (95% 13.9–23.9) among women (249 people were examined) [61]. A high seroprevalence was noted in Pakistan. In the country as a whole, it was 42.4% (41.5–43.14). Among men, it was 42.8% (41.9–43.7); among women, it was 40.5% (38.7–42.2) [67]. These selective data confirm the position of Chen [47] about a significant range of SARS-CoV-2 seroprevalence in different regions of the world.

Unfortunately, works from Russian authors have not yet found full reflection in the English-language literature [47]. Considering that Russia occupies one-eighth of the world’s land mass, this lack of information in the global literature reduces global data accuracy. Perhaps the publication of this English version of the work will help to fill in information gaps to some extent. Further, it is worth mentioning that a large group of authors have already published 16 articles in Russian publications under the auspices of the head of Rospotrebnadzor, Professor A. Y. Popova [17,18,19,22,23,24,25,26,27,28,29,30,31,32,33].

In Russia, the average seroprevalence for all FDs was 19.2% (95% CI 19.0–19.6), varying from 9.8% (95% CI 8.6–11.0) in the Siberian FD up to 31.9% (95% CI 30.9–32.9) (Table 1). No differences were found by gender: seroprevalence among men was 16.95% (IQR: 9.4–21.9); among women, it was 18.4% (IQR: 9.7–25.1). In general, the seroprevalence in Russia is 3.5-fold higher than the world average [9], yet more than two-fold lower than in Qatar and Pakistan [62,67]. The question arises: What is the reason for these differences? One of the reasons may be the type of Ag used in serological studies. The receptor binding domain (RBD) or Nc Ags are the most commonly used for this purpose. In addition, the question of Ag choice for sero-diagnostics is not trivial. In response to RBD, the body generates neutralizing antibodies, while the neutralizing activity generated in response to the Nc Ag is lower [57]. Ab to Nc are more indicative of infection, but not of neutralizing activity.

As already noted, a feature of seroprevalence in Russia is a significant dispersion of data (Table S3). Among the regions where seroconversion approached the 50% mark were Kaliningrad region (50.2% (95% CI 48.4–52.0)) and Amur region (45.4% (95% CI 43.6–47.2)). Regions with minimal seroprevalence were also identified, such as the Republic of Crimea (4.3% (96% CI 3.6–5.1)) and Irkutsk region (5.8% (95% CI 4.9–6.7)). In trying to find the reasons for such a significant difference, we drew attention to the climate and geographic factors. All of the surveyed regions are located practically in four main climatic zones: northern subtropics (Republic of Crimea, Krasnodar region); middle zone (Moscow city and the Moscow, Belgorod, Vladimir regions); coastal territories (Primorsky, Amur, Kaliningrad, and Leningrad regions, St. Petersburg city); and northern regions with a sharply continental climate (Irkutsk, Novosibirsk, Krasnoyarsk and Murmansk regions, located in the zone of the Far North).

In latitudinal terms, the regions are located in the territory from 45.02742 to 68.95852 degrees northern latitude. Such a large latitudinal extent also explains climatic differences. Hence, the average annual temperature in the southern part of Crimea is +3.9 °C, and the average annual temperature in the north of the Murmansk region is 0.1–2.3 °C, while the average annual temperature in the north of the Krasnoyarsk region is even lower than 0.1 °C. In this regard, we tried to assess the influence of climato-geographic factors on the level of collective immunity to SARS-CoV-2. The analysis revealed a weak correlation with a level of reliability of r = 0.33 (*p* ≥ 0.1), described by a linear regression equation (y = 0.653x − 15.953); the coefficient of determination (R^2^) was 0.1 (Figure 1). The relationship is undoubtedly weak and is one of many factors, among which subject age may play a more significant role.

In the process of stratification of volunteers by age, the largest proportion of seropositive cases was revealed in persons aged 1–17 years (21.6% (IQR: 13.1–31.7)). This result is consistent with other data [9]. These results are quite consistent with accepted opinion about a greater resistance of children to COVID-19, often with mild or asymptomatic forms [68]. There was no evidence of increased seroprevalence among children in Israel [69]. However, the high level of collective immunity achieved in that country, as a result of active vaccination, probably erased any existing differences.

As for the seroprevalence in volunteers in other age groups, no statistically significant differences were found between them (Table S3). More than 75% of all seroprevalence values in the studied cohort are within the IQR (Figure 4).

**Figure 10:**
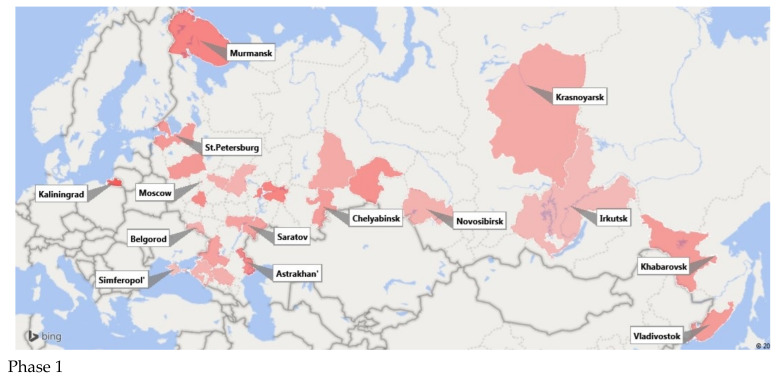

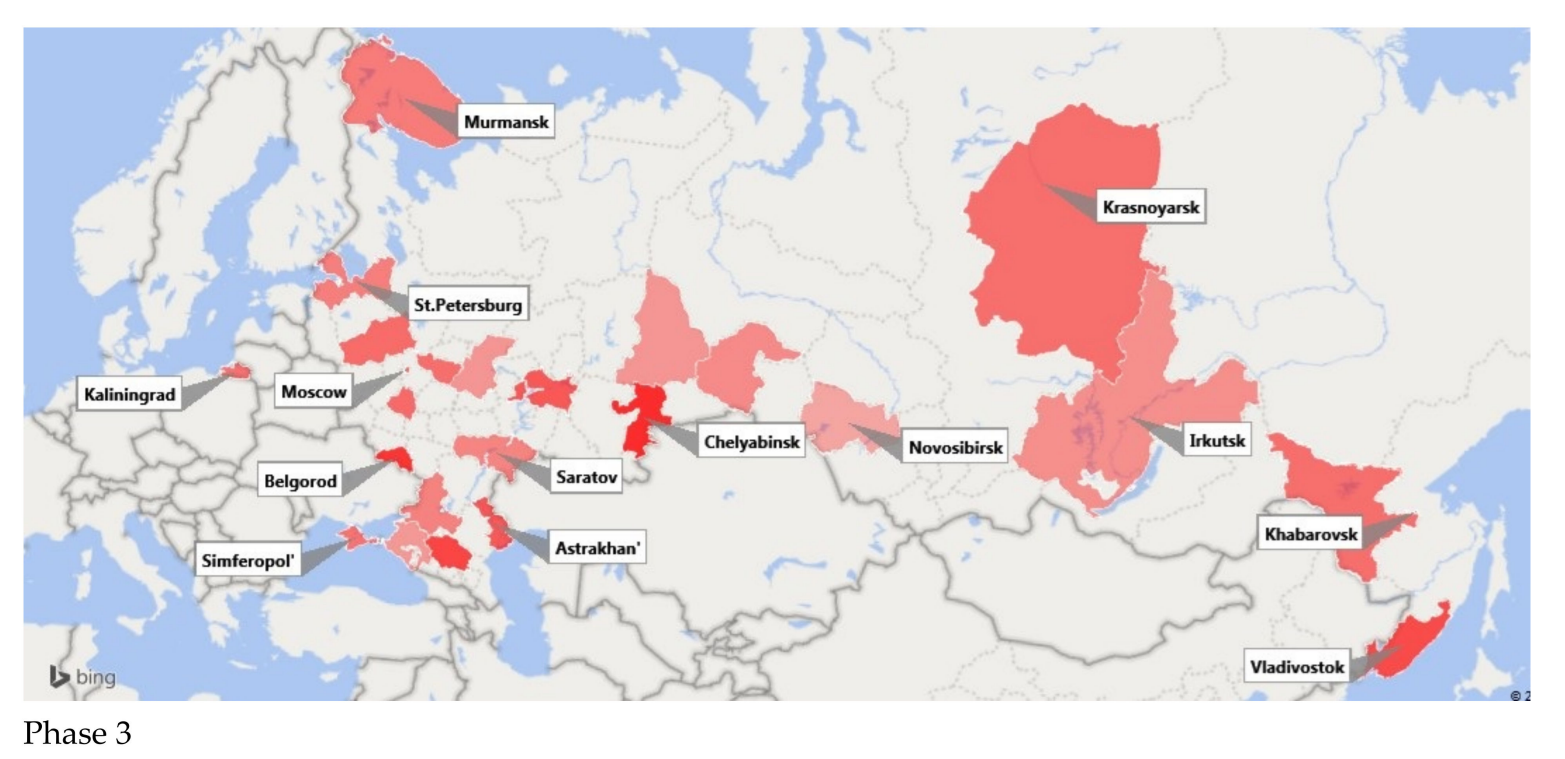
Color intensity representation of SARS-CoV-2 seroprevalence, Russian population, 2020. Phase 1—Seroprevalence levels in the first phase of monitoring (June–August). Phase 3—levels in the third phase (December). Color intensity reflects the percent seropositive: the more intense the color, the higher the level of population immunity.

One piece of the project’s design was a longitudinal, three-phase study of SARS-CoV-2 seroprevalence in volunteers during natural development of the COVID-19 epidemic. In 2020, three study phases were carried out: a first phase (June–July); a second phase (September to October); and a third phase (December). According to incidence data the first two phases occurred at the end of the epidemic’s first wave during a period of relative calm, and the third phase was carried out at the peak of the second wave’s morbidity. The dynamics of seroprevalence changed accordingly (Figure 5). Hence, the weighted average seroprevalence values were: first phase with 18.6% (IQR: 10.7–23.0); second phase with 27% (IQR: 17.3–38.0); and third phase with 39.7% (IQR: 31.0–43.8). The data differences between the first and third phases are statistically significant (*p* < 0.05). The results are represented graphically in Figure 10.

Thus, the third, highest level of population immunity was formed by the end of the second wave of COVID-19 (https://coronavirus-monitor.info/country/russia/, accessed on 10 June 2021) Note that, at the 2020–2021 transition, there was a trend reversal from upward to downward, which could be interpreted as the completion of the second COVID-19 wave. The downtrend persisted almost until the end of May 2021, after which another trend change and a new exacerbation of infection were noted in early June (2021). This validates the existing idea of an undulating course with respiratory infection epidemics, including COVID-19.

In the process of sero-epidemiological examination of the population, three main groups were distinguished: convalescent; contact; and PCR-positive. Regarding the last group, these are the strangest, i.e., patients who do not have other illness symptoms, but can be carriers and distributors of the virus within the context of an asymptomatic COVID-19 course.

As for convalescents, the proportion seropositive among them significantly exceeded the average for the region (57.3% (IQR: 38.6–72.3)). The role of convalescents is not limited to a contribution to collective immunity, as evidenced by a high correlation between the general seropositivity in the cohort, and specifically among convalescents (Figure 7). A similar relationship was seen between the number of convalescents and persons who had verified contact with a sick or recovering COVID-19 patient. The arithmetic ratio of these two categories of surveyed volunteers was 6.0 (4.3–8.5), which indicates that one COVID-19 patient or convalescent, in theory, can infect at least six healthy, seronegative people [7,70]. Some of these individuals will develop a manifest form of disease; others will join the group ‘PCR+ subjects’ and their further fate is unknown. A third category will replenish the seropositive share of surveyed volunteers, as evidenced by a high correlation between the number of seropositive and contact persons (Figure 9); some of them, of course, will be in the group ‘asymptomatic seropositive volunteers’, whose number in the cohort reached 93.6% (IQR: 87.1–94.9). The size of this group, the least understood in terms of outcome, may, according to some sources, vary from 6% to 96%.

## 4. Conclusions

Here for the first time, a longitudinal three-phase study of collective immunity to SARS-CoV-2 in the Russian population was carried out. The study was conducted from June to 31 December 2020. It was found that the total proportion of persons with Ab to SARS-CoV-2 Nc, in the initial phase of the study, varied within the range 18.6% (IQR: 10.7–23.0). By the end of the third phase of monitoring, seroprevalence had increased to 39.7% (IQR: 31.0–43.8). The results obtained allowed us to identify a significant correlation between the number of convalescents and contact persons; this may indicate a significant role of convalescents in the spread of SARS-CoV-2. These sero-monitoring results may serve as a prerequisite for further research aimed at controlling the COVID-19 pandemic.

## Figures and Tables

**Table 1:** Distribution of model regions by Russian Federal District [17,18,19,22,23,24,25,26,27,28,29,30,31,32,33].

Federal District(FD)	Number of Regions in the FD	Total number of Population Constituents	Characteristics of the Surveyed Regions in the FD			Characteristics of the Surveyed Cohorts		
			Number of Surveyed Regions in the FD	Population Size of Surveyed Areas, Persons	Also in %	Number of volunteers	Number of Seropositive Persons	Seroprevalence, % (95% Confidence Interval, CI)
FEFD	11	8,124,053	3	3,960,816	48.8	8295	2384	28.7 (27.8–29.7) *
NCFD	7	9,967,301	1	2,792,796	28.0	2683	262	9.8 (8.6–11.0) *
UFD	6	12,329,500	3	9,276,273	752	8584	1589	18.5 (17.6–19.4)
NWFD	11	13,941,959	4	9,028,541	64.8	11,899	3800	31.9 (30.9–32.9) *
SbFD	10	17,003,927	3	8,016,756	47.1	8165	748	9.2 (8.3–9.7) *
SFD	8	16,482,488	4	12,764,789	77.4	11,632	1633	14.0 (13.4–14.7) *
VFD	14	29,070,827	3	9,465,793	32.6	8982	1695	18.9 (17.9–19.8)
CFD	18	39,250,960	5	24,706,022	63.9	13,964	2164	15.7 (15.1–16.5) *
Total	85	146,171,015	26	80,011,786	54.8	74,158	14,275	19.2 (19.0–19.6)

Notes. Abbreviations: FEFD—Far Eastern Federal District; NCFD—North Caucasian Federal District; UFD—Ural Federal District; NWFD—Northwestern Federal District; SbFD—Siberian Federal District; SFD—Southern Federal District; VFD—Volga Federal District; CFD—Central Federal District. * Statistically significant differences compared to the mean (*p* < 0.05). Sources: http://www.demoscope.ru/weekly/knigi/ns_r01/pril_1.html; http://www.statdata.ru/naselenie-federalnyh-okrugov-rossii; https://en.wikipedia.org/wiki/List_of_federal_subjects_of_Russia_by_population (Accessed: 9 June 2021).

## Data Availability

The authors confirm that the data supporting the findings of this study are available within the article and/or its supplementary materials.

## Supplementary Materials

The following are available online at https://www.mdpi.com/article/10.3390/v13081648/s1, Table S1: Region and age related volunteer cohort patterns [17]. Table S2: Volunteer numbers, by administrative region. Table S3: Distribution of (SARS-CoV-2) seropositive volunteer proportions. Table S4: Distribution of seropositive, asymptomatic volunteers by age and area of residence.
