# Supplementary material for: SARS-CoV-2 Seroprevalence Structure of the Russian Population during the COVID-19 Pandemic
Source: Viruses. 2021 Aug 19;13(8):1648. doi: 10.3390/v13081648 (PMC8402751; doi:10.3390/v13081648)
Supplement: Supplementary file 1 [file viruses-13-01648-s001.zip › viruses-1299301-si final.pdf]

Supplementary materials

Table S1. Region and age related volunteer cohort patterns [1].

Region	Number of volunteers examined	Sample size in various age groups (yrs). individuals						
		1-17	18-29	30-39	40-49	50-59	60-69	70+
Amur region	2936	385	392	427	456	458	452	366
Astrakhan Region	2689	286	385	391	376	386	381	384
Belgorod Region	2806	392	383	387	396	389	401	458
Vladimir Region	2798	302	225	585	714	584	336	52
Irkutsk Region	2674	387	385	373	374	386	390	379
Kaliningrad Region	2939	317	395	465	488	493	465	316
Krasnodar Territory	2999	427	366	363	460	494	452	437
Krasnoyarsk Territory	2807	402	409	440	441	449	420	246
Leningrad Region	3130	401	464	474	510	480	422	379
Moscow	2688	384	384	384	384	384	383	385
Moscow Region	2688	384	384	384	384	384	384	384
Murmansk Region	3117	378	429	478	510	517	417	388
Nizhny Novgorod Region	2687	266	367	495	481	439	337	302
Novosibirsk Region	2728	388	399	388	391	409	376	377
Primorsky Territory	2684	380	384	384	384	384	384	384
Republic of Crimea	2896	314	447	563	407	451	336	378
Rostov Region	3048	404	394	453	512	440	453	392
St. Petersburg	2713	377	390	416	395	413	442	280
Saratov Region	3369	409	474	653	634	541	432	226
Sverdlovsk Region	3149	404	438	515	431	493	461	407
Stavropol Territory	2683	383	379	385	384	383	384	385
Republic of Tatarstan	2926	400	400	448	461	448	382	387
Tula Region	2894	426	392	436	424	424	409	383
Tyumen Region	2758	356	485	452	447	482	387	149
Khabarovsk Territory	2675	383	385	383	382	382	380	380
Chelyabinsk Region	2677	370	344	406	413	382	390	372
Total	74 158	9705	10 279	11 528	11 639	11 475	10 456	8976

Table S2. Volunteer numbers. by administrative region.

Region	Primary number. people	Number of volunteers in each phase		
		1st phase	2nd phase	3rd phase
Amur region	2936	2116	1973	2116
Astrakhan region	2689	1263	1170	1263
Belgorod region	2806	1722	1623	1722
Chelyabinsk region	2677	1371	1076	1371
Irkutsk region	2674	1940	1491	1938
Kaliningrad region	2939	2036	1702	2036
Khabarovsk territory	2675	1610	1312	1687
Krasnodar Territory	2999	1987	1588	1990
Krasnoyarsk territory	2807	1809	1594	1806
Leningrad region	3130	1744	1455	1743
Moscow city	2688	385	641	642
Moscow region	2688	1252	1078	1251
Murmansk region	3117	1750	1539	1750
Nizhny Novgorod Region	2687	1314	1055	1315
Novosibirsk region	2728	1651	1723	1651
Primorsky territory	2684	1468	1151	1467
Republic of Crimea	2896	1452	1090	1450
Republic of Tatarstan	2926	2858	2858	2858
Rostov region	3048	2010	1587	2012
Saratov region	3369	1502	1077	1500
St. Petersburg city	2713	1791	1518	1791
Stavropol Territory	2683	1011	900	1011
Sverdlovsk region	3149	2044	1548	2044
Tula region	2894	1800	1591	1800
Tyumen region	2758	1900	1555	1900
Vladimir region	2798	1215	863	1215
Total	74158	43396	36304	42426

Table S3. Distribution of (SARS-CoV-2) seropositive volunteer proportions.

Region	Percent seropositive volunteers. by age (years) (M. 95% CI. %)								
	1-17	18-19	20-29	30-39	40-49	50-59	60-69	70+	Итого по когорте
Amur region	61.6*<br>56.5-66.4	49.2<br>44.2-54.3	36.3*<br>31.7-41.1	36.3*<br>31.7-41.1	40.1<br>35.6-44.8	41.5<br>36.9-46.2	45.6<br>40.9-50.3	45.9<br>40.7-41.2	45.4*<br>43.6-47.2
Astrakhan region	43*<br>38.0-48.1	20.3**<br>16.4-24.6	23.3<br>19.2-27.8	23.3<br>19.2-27.8	22.1<br>17.9-26.6	19.7*<br>15.8-24.0	17.6*<br>13.9-21.8	45.1<br>40.0-50.2	27.3*<br>26.7-30.2
Belgorod region	10.7<br>8.5-16.1	7.3<br>4.9-10.4	7.5<br>5.1-10.6	7.5<br>5.1-10.6	10.6<br>7.8-14.1	8.2<br>5.7-11.4	9.5<br>6.8-12.8	7.2<br>5.0-9.9	8.4*<br>7.4-9.5
Vladimir region	19.9<br>15.5-24.8	7.5<br>4.5-11.8	8.7<br>6.6-11.3	8.7<br>6.6-11.3	8.4<br>6.5-10.7	11.6<br>9.2-14.5	9.8<br>6.9-13.5	19.2<br>9.6-32.5	10.7<br>9.6-11.9
Irkutsk region	8<br>5.5-11.2	6.8<br>4.5-9.7	4<br>2.3-6.6	4<br>2.3-6.6	4.3<br>2.5-6.9	4.4<br>2.6-6.9	3.6<br>1.9-5.9	9.2<br>6.5-12.6	5.8*<br>4.9-6.7
Kaliningrad region	66.9*<br>61.4-72.0	57<br>51.9-61.9	47.3<br>42.7-1.9	47.3<br>42.7-51.9	51<br>46.5-55.5	44.2<br>39.8-48.7	46<br>41.4-50.7	43.4<br>37.8-49.0	50.2*<br>48.4-52.0
Krasnodar Territory	6.8<br>4.6-9.6	5.7<br>3.6-8.6	5.8<br>3.6-8.7	5.8<br>3.6-8.7	5.0<br>3.2-7.4	6.7<br>4.6-9.3	7.3<br>5.1-10.1	18.3<br>14.8-22.3	8*<br>7.1-9.0
Krasnoyarsk territory	13.2<br>10.0-16.9	15.2<br>11.8-19.0	9.8<br>7.2 -12.9	9.8<br>7.2 - 12.9	11.1<br>8.3 - 14.4	11.4<br>8.6 - 14.7	13.3<br>10.2-16.9	13.4<br>9.4-18.3	12.4<br>11.2 - 13.6
Leningrad region	26.9*<br>22.7-31.6	17.7<br>14.3-21.5	20.5<br>16.9-4.4	20.5<br>16.9-24.4	17.2<br>14.1-20.8	17.7<br>14.4-21.4	18.5<br>14.9-22.5	29.0<br>24.5-33.9	20.7<br>19.3-22.2
Moscow city	37*<br>32.1-42.0	15.9*<br>12.4-19.9	16.7<br>13.1-20.8	16.7<br>13.1-20.8	17.2<br>13.6-21.3	23.2<br>19.1-27.7	24.5<br>20.3-29.2	20.5<br>6.6-24.9	22.1<br>20.6-23.8
Moscow region	26<br>21.7-30.7	16.4<br>12.8-20.5	21.6<br>17.6-26.1	21.6<br>17.6-26.1	22.1<br>18.1-26.6	22.1<br>18.1-26.6	21.1<br>17.1-25.5	17.7<br>14.0-21.9	21<br>19.5-22.6
Murmansk region	37.3<br>32.4-42.4	37.1<br>32.5-41.8	40.2<br>35.7-44.7	40.2<br>35.7-44.7	34.7<br>30.6-39.0	26.5<br>22.7-30.5	20.4<br>16.6-24.6	20.9<br>16.9-25.3	31.2*<br>29.6-32.8
Nizhny Novgorod Region	10.2<br>6.8-14.4	6<br>3.8-8.9	7.5<br>5.3 -10.2	7.5<br>5.3 - 10.2	9.8<br>7.3 - 12.8	8.4<br>6.0 -11.4	9.5<br>6.6 - 13.1	7.6<br>4.9-11.2	8.4*<br>7.4 - 9.5
Novosibirsk region	13.1<br>9.9-16.9	5.5<br>3.5-8.2	4.9<br>2.9-7.5	4.9<br>2.9-7.5	9.7<br>6.9-13.1	7.6<br>5.2-10.6	8.0<br>5.5-11.2	14.9<br>11.4-18.9	9*<br>8.0-10.2
Primorsky territory	23.9	13.3*	17.4	17.4	22.4	21.9	19.5	19 1	19.6

	19.7-28.6	10.1-17.1	13.8-21.6	13.8-21.6	18.3-26.9	17.8-26.4	15.7-23.9	5.2-23.3	18.2-21.2
Republic of Crimea	2.5<br>1.1-4.9	5.4<br>3.5-7.9	4.4<br>2.9-6.5	4.4<br>2.9-6.5	5.9<br>3.8-8.7	5.3<br>3.4-7.8	4.2<br>2.3-6.9	1.6<br>0.6-3.4	4.3*<br>3.6-5.1
Republic of Tatarstan	39.2*<br>34.4-44.2	32.2<br>27.7-7.1	30.6<br>26.3-35.1	30.6<br>26.3-35.1	28.4<br>24.3-32.8	34.4<br>29.9-38.9	31.7<br>27.0-36.6	24<br>19.9-28.6	31.5 *<br>29.8-33.2
Rostov region	30.7*<br>26.2-35.4	13.5<br>10.2-17.2	12.8<br>9.9-16.2	12.8<br>9.9-16.2	16.0<br>12.9-19.5	14.5<br>11.4-18.2	16.3<br>13.1-20.1	12.5<br>9.4-16.2	16.5<br>15.2-17.9
St. Petersburg city	32.1<br>27.4-37.1	21.3<br>17.3-25.7	22.8<br>18.9-27.2	22.8<br>18.9-27.2	22.3<br>18.3-26.7	25.4<br>21.3-29.9	29<br>24.8-33.4	30.4<br>25.0-36.1	26*<br>24.3-27.7
Saratov region	18.1<br>14.5-22.2	18.8<br>15.4-22.6	15.5<br>12.8-18.5	15.5<br>12.8-18.5	14.5<br>11.9-17.5	16.8<br>13.8-20.2	15.7<br>12.4-19.5	14.6<br>10.3-19.9	16.3<br>15.0-17.6
Sverdlovsk region	17.8*<br>14.2-21.9	11.9<br>9.0-15.3	12.8<br>10.1-16.0	12.8<br>10.1-16.0	7.9<br>5.5-10.9	11.6<br>8.9-14.7	11.1<br>8.4-14.3	14.7<br>11.4-18.6	12.4<br>11.3-13.7
Stavropol Territory	17.0*<br>13.4- 21.1	9.5<br>6.7 -12.9	8.1<br>5.5 - 11.2	8.1<br>5.5 - 11.2	4.7<br>2.8 - 7.3	8.6<br>6.0 - 11.9	9.9<br>7.1 - 13.3	10.6<br>7.8 - 14.2	9.8<br>8.7-10.9
Tula region	11.3*<br>8.4-14.7	12.8<br>9.6-16.5	13.3<br>10.3-16.9	13.3<br>10.3-16.9	15.1<br>11.8-18.9	15.3<br>12.0-19.1	21.8<br>17.9-26.1	22.7<br>18.6-27.2	15.9<br>14.6-17.3
Tyumen region	26.4<br>21.9-31.3	25.8<br>21.9-29.9	26.3<br>22.3-30.6	26.3<br>22.3-30.6	24.2<br>20.3-28.4	26.1<br>22.3-30.3	22<br>17.9-26.4	13.4<br>8.4-19.9	24.5<br>22.9-26.2
Khabarovsk territory	23.0<br>18.9- 7.5	19.0<br>15.2-23.2	15.4<br>11.9 -19.4	15.4<br>11.9-19.4	14.7<br>11.3-18.6	20.9<br>16.9- 25.4	21.8<br>17.8- 26.3	22.6<br>18.5- 27.2	19.6<br>18.1 - 21.2
Chelyabinsk region	20.3<br>16.3-24.7	27.0<br>22.4-32.1	18.2<br>14.6-22.3	18.2<br>14.6-22.3	20.1<br>16.3-24.3	18.3<br>14.6-22.6	17.7<br>14.0-21.9	17.7<br>14.0-21.6	19.4<br>17.9-20.9
Median	21.6	15.6	15.6	15.45	15.6	17.2	17.7	18.0	17.95
IQR	13.1 - 31.7	8 - 21.1	8- 21.1	8.2 - 22.6	9.7 - 22.2	9.3- 22.9	9.8- 21.8	13.4- 22.6	10.0 - 23.9

Key: * - differences are statistically significant (more or less than the median values in the general cohort) ($p<0.05$). IQR - interquartile range (Q25 – Q75).

Table S4. Distribution of seropositive. asymptomatic volunteers by age and area of residence.

Region	Distribution of seroprevalence by age. years M±m							Total in region
	1-17	18-29	30-39	40-49	59-59	60-69	70+	
Amur region	92.0±1.8	91.2±2.0	89.7±2.4	95.6±1.5	95.3±1.5	96.6±1.3	98.8±0.8	94.1±0.6
Astrakhan region	81.9±2.9	85.9±3.9	81.3±4.1	74.7±4.8	76.3±4.9	98.5±1.5	95.4±1.6	85.6±1.3
Belgorod region	97.6±2.4	89.3±5.8	96.6±3.4	92.9±4.0	87.5±5.8	97.4±2.6	97.0±3.0	94.3±1.5
Vladimir region	95.0±2.9	82.4±9.2	96.1±2.7	86.7±4.4	85.3 ±4.3	75.8 ±7.5	70.0±14.5	87.6 ±2.0
Irkutsk region	96.8±3.1	69.2±9.0	53.3±13	62.5±12.1	70.6±11.0	85.7±9.4	100	81.2±3.2
Kaliningrad region	96.7±1.2	94.7±1.5	95.5±1.4	94.4±1.4	94.0±1.6	95.3±1.4	96.4±1.6	95.2±0.6
Krasnodar Territory	100	100	95.2±4.6	100	100	93.9±4.2	100	98.8±0.7
Krasnoyarsk territory	94.3±2.3	91.9±2.5	97.7±1.6	87.8±3.4	94.1±2.4	94.6±2.2	93.3±3.1	93.4±0.9
Leningrad region	92.6±4.9	89.0±6.8	84.5±7.2	89.8±6.3	82.4±8.1	80.8±8.7	87.3±6.2	86.9±2.6
Moscow city	86.6±2.9	91.8±3.5	78.1±5.2	84.8±4.4	65.2±5.1	85.1±3.7	84.8±4.0	82.4±1.6
Moscow region	92.0±1.9	87.3±3.1	84.3±2.9	81.2±3.1	77.6±3.4	76.5±3.5	83.8±3.3	83.4±1.1
Murmansk region	92.9±2.2	92.5±2.1	89.1±2.3	89.3±2.3	92.0±2.3	74.1±4.8	85.2±4.0	89.0±1.0
Nizhny Novgorod Region	92.6±3.6	86.4±5.3	86.5±4.1	89.4±3.2	70.3±5.8	100	100	88.4±1.5
Novosibirsk region	98±2.0	100	89.5±7.0	94.7±3.6	90.3±5.3	93.3±4.5	94.6±3.0	94.7±1.4
Primorsky territory	100	98.0±2.0	98.5±1.5	98.8±1.2	98.8±1.2	93.3±2.9	100	98.3±0.6
Republic of Crimea	100	95.8±4.1	100	95.8±4.1	100	93.0±6.8	100	97.6±1.4
Republic of Tatarstan	98.7±0.9	97.7±1.3	90.5±2.5	91.6±2.4	90.9±2.3	93.4±2.3	100	94.5±0.8
Rostov region	99.2±0.8	96.2±2.6	98.3±1.7	93.9±2.6	95.3±2.6	98.6±1.4	98.0±2.0	97.2±0.7
St. Petersburg city	85.9±3.2	81.9±4.2	91.6±2.8	88.6±3.4	80.0±3.9	82.0±3.4	82.4±4.1	84.5±1.4
Saratov region	91.9±3.2	87.6±3.5	96.0±1.9	87±3.5	90.1±3.1	91.2±3.4	97±2.9*	91.1±1.2
Sverdlovsk region	91.7±3.2	92.3±3.7	89.4±3.8	100	100	96.1±2.7	95.0±2.8	94.4±1.2
Stavropol Territory	90.8±3.6	97.2±2.7	100	94.4±5.4	93.9±4.2	86.8±5.5	85.4±5.5	92.0±1.7
Tula region	95.8±2.9	94.0±3.4	74.1±5.8	82.8±4.7	78.5±5.1	80.9±4.2	95.4±2.2	85.7±1.6
Tyumen region	98.9±2.1	95.2±3.8	96.6±3.3	100.0±1.9	97.6±2.7	98.8±2.3	100	97.8±1.1
Khabarovsk territory	94.3±4.8	95.9±4.6	91.5±7.1	98.2±3.5	88.8±6.9	94.0±5.1	94.2±4.9	93.7±2.1
Chelyabinsk region	94.7±2.6	94.6±2.3	94.6±2.6	94.0±2.6	97.1±2.0	91.3±3.4	100	95.0±0.9
median (Me)	94.5	92.4	91.6	92.2	90.6	93.3	95.8	93.6
IQR (Q_{25} : Q_{75})	92-97.9	88.0-95.9	87.2-96.4	87.2-95.5	80.6-95	85.2-95.9	88.8-100	87.1-94.9

Note. Given the predominance of mean values close to 100%, we replaced the calculation of the 95% CI with the calculation of the mean percentage error.

Supplementary References

1. Popova, A.Y.; Andreeva, E.E.; Babura, E.A.; Balakhonov, S.V.; Bashketova, N.S.; Bulanov, M.V.; Valeullina, N.N.; Goryaev, D.V.; Detkovskaya, N.N.; Ezhlova, E.B.; et al Features of developing SARS-CoV-2 nucleocapsid protein population-based seroprevalence during the first wave of the COVID-19 epidemic in the Russian Federation. *Russ. J. Infect. Immun. [Infektsiya I Immun.]* **2021**, *11*, 297–323. (In Russian) doi:10.15789/2220-7619-FOD-1684.
